# A Conjugate Thermo-Electric Model for a Composite Medium

**DOI:** 10.1371/journal.pone.0097895

**Published:** 2014-05-27

**Authors:** Oscar Chávez, Francisco A. Godínez, Alberto Beltrán, Armando García, Roberto Zenit

**Affiliations:** 1 Instituto de Investigaciones en Materiales, Universidad Nacional Autónoma de México, Ciudad Universitaria, D.F., México; 2 Instituto de Ingeniería, Universidad Nacional Autónoma de México, Ciudad Universitaria, D.F., México; 3 Tecnología Aplicada en Exploración y Producción Petrolera, Inc., Polanco, D.F., México; Queen's University at Kingston, Canada

## Abstract

Electrical transmission signals have been used for decades to characterize the internal structure of composite materials. We theoretically analyze the transmission of an electrical signal through a composite material which consists of two phases with different chemical compositions. We assume that the temperature of the biphasic system increases as a result of Joule heating and its electrical resistivity varies linearly with temperature; this last consideration leads to simultaneously study the electrical and thermal effects. We propose a nonlinear conjugate thermo-electric model, which is solved numerically to obtain the current density and temperature profiles for each phase. We study the effect of frequency, resistivities and thermal conductivities on the current density and temperature. We validate the prediction of the model with comparisons with experimental data obtained from rock characterization tests.

## Introduction

A composite medium can be defined as that made of at least two phases of different chemical compositions [1]. The study of composite media are of great interest to various areas such as physics, chemistry and materials science, among others [1]–[3].

The response of composite media when transmitting-absorbing waves of different intensities and frequencies has been analyzed by many previous studies. In [4], a fiber reinforced epoxy matrix composite is studied as electromagnetic wave absorbing material in a wide frequency range. Carbon fiber reinforced concrete [5] and metallic wire structures [6] have been characterized in terms of their capacity to absorb electromagnetic fields. It is interesting to note that some materials have the ability to shield electromagnetic waves, examples of these are styrene-butadiene rubber composites [7], wood-cement boards [8] and nanocomposites [9].

The prediction of the effective properties of a composite medium is commonly based on the knowledge of both the volume fraction distribution and the value of the property of each of its phases [1]. A series of mathematical models have been developed to define global properties of a composite medium. In [1], [10] the effective electrical conductivity is obtained from the individual electrical properties, also, heat transfer studies at macroscopic and microscopic levels have been conducted to determine the effective thermal conductivity [11], [12]. To our knowledge the interaction between the heat diffusion and transmission of electric current through composite media has not been studied fully to date.

**Figure 1:**
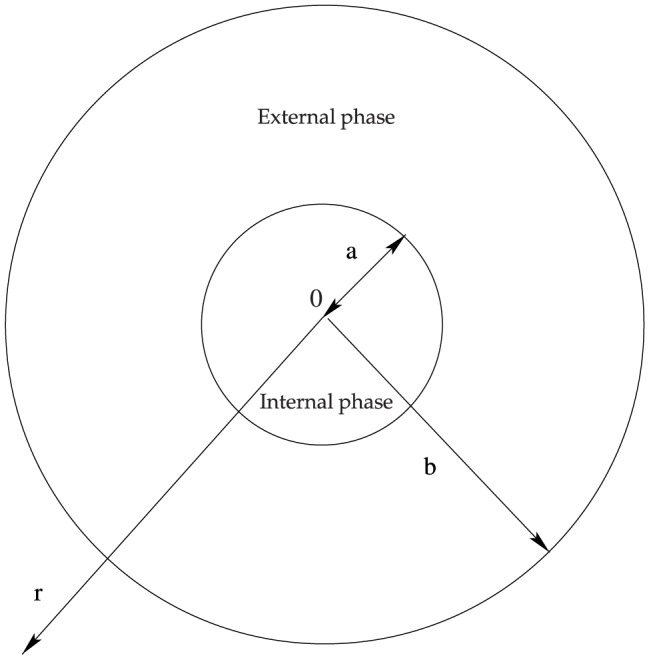
Schematic representation of a composite medium.

The present theoretical model considers two non-deformable phases which are modeled as continua, and it is based on a previous model developed by Chávez and Méndez [13] who analyzed the conjugate heat and electromagnetic transfer mechanism in a bimetallic conductor. Our system consists of a cylindral external phase confining a second cylindrical internal phase, henceforth external and internal phases, as depicted schematically in Figure 1.

Maxwell's equations are coupled with the heat conduction equation considering a heat source term to account for Joule's effect. This coupling is performed for each phase; also, both phases are coupled with each other through the boundary conditions at the common interface.

Particularly, the proposed model is intended to explain the role of some effects that occur during the electrical conduction processes in composite media, which to our knowledge have not been fully addressed:

1. The so-called skin effect observed in power transmission lines [14].
2. The Joule heating effect [15].
3. The effect of frequency on the current density and temperature distribution.
4. The effect of volumetric fraction (porosity) on the bulk electrical resistivity.

## Modelling

The physical model under study is a composite medium like that shown in Figure 1. We consider an external phase with radius 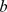 and an internal one with radius 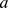. A sudden alternating electric current through this biphasic conductor is established. Thus, a rise of temperature results from the flow of electric current, caused by Joule's effect. We assumed that the resistivity in both phases varies linearly with temperature. For large values of the frequency associated to the alternating current, a redistribution of the current density is inevitable and the skin effect yields a tendency of the electric current to flow over the outer surfaces of both phases. In real conditions, the current density distribution depends on the values of electrical resistivities of each phase and the heat generated by Joule's effect which is transferred by heat conduction. Finally, we model the heat transferred to the environment by a convection process.

### Electromagnetic model

Using Maxwell's equations, we can readily derive a wave equation to analyze the electromagnetic propagation. Therefore, the current density is governed by the following equation [16]: 
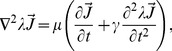
(1)where, 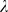 is the electric resistivity, 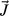 is the current density, 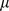 is the magnetic permeability, 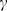 is the electric permitivity and 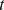 is the physical time.

We consider only variations of the current density in the radial direction and the alternating current behaves like a sinusoidal wave. Therefore, the current density can be written as 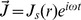. On the other hand, the electrical resistivity has a linear variation with temperature [16] which can be written as 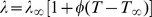, and introducing it into Eq. (1) we obtain that, 
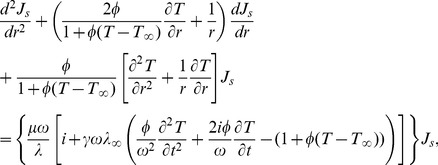
(2)where 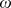 is the frequency of the electrical signal, 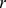 is the radial coordinate, 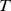 is the temperature, 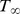 is the environment temperature, 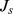 is the current density function depending only on radial coordinate, 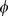 is the temperature coefficient for resistivity and 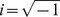.

In most practical cases, the term 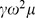 is smaller than 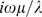 and can be neglected as a first approximation. In addition, we also introduce the well-known conductor skin depth parameter, 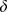, defined by 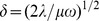 [17]. Thus, Eq. (2) can be rewritten for the internal phase as: 
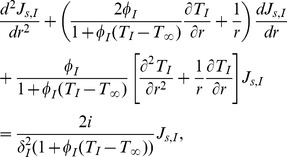
(3)and for the external phase, 
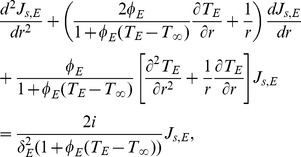
(4)where the subscript “
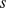
” is used to denote the spatial dependence and the subscripts “
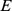
” and “
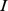
” are used to denote external and internal phases, respectively. The “
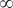
” subscript refers to external environmental conditions.

The above equations system must be solved considering the following boundary conditions: 
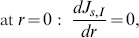
(5)

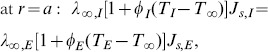
(6)

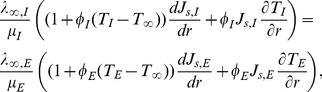
(7)

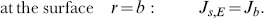
(8)


The boundary condition at the centre (Eq. (5)) is the symmetry condition, while the continuity of the electric field at the interface is expressed by Eq. (6). Eq. (7) refers to the continuity of the magnetic field, while Eq. (8) expresses a characteristic current density. 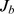 is the current density at the outer surface of the external phase and should be determined with the following restriction: 
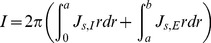
(9)


### Thermal model

The general heat diffusion equation can be expressed as [18]: 
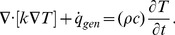
(10)


With this equation we can determine the gradients of temperature in both phases by taking into account their thermal properties and the amount of heat generated on each of them.

Equation (10) is simplified by considering only temperature variations in the radial direction and exclusively heat generation by Joule's effect. Therefore, for the internal phase we have: 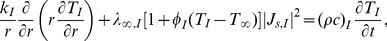
(11)and for the external phase: 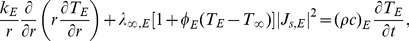
(12)subjected to the following boundary conditions: 
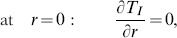
(13)

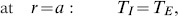
(14)

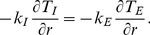
(15)


For the outer surface of the external phase, we have 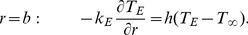
(16)


Also an initial condition is necessary. Here we have considered that the composite media is initially at ambient temperature: 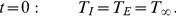
(17)


In the above equations, 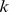 is the thermal conductivity, 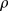 is the density, 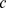 is the specific heat, 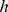 is the convective heat transfer coefficient.

## Dimensional Analysis

In order to reduce the number of physical parameters, we can perform a dimensional analysis. We first identify the characteristic convective time scale 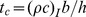. On the other hand, the suitable spatial scale is chosen as the radius of the external phase, 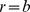. Furthermore, the characteristic temperature drop 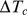 can be obtained through an energy balance between the heat generation term and the transient term, i.e.:
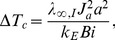
(18)where 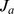 is the current density at the surface of the internal phase and 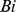 is the Biot number which measures the environmental conditions and is defined as 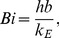
(19)


With the above set of characteristic geometrical and physical scales, the electromagnetic and thermal models can be simplified by introducing the following dimensionless variables and parameters:
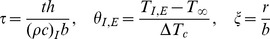


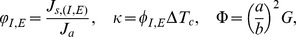


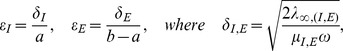
where 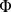 is the volumetric fraction defined as the ratio of the volume of the internal phase and the total volume of the composite media, while 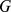 is defined as the actual lenght of the inner path divided by the straight-line distance between the ends of the inner path, for porous media applications this parameter is known as tortuosity and is always larger or equal than one.

### Dimensionless electromagnetic model

The system (3)–(8) can be rewritten in dimensionless form by using the above dimensionless parameters and variables.

The internal phase dimensionless model is: 
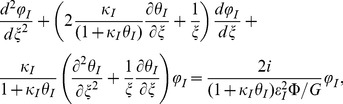
(20)and external phase dimensionless model is: 
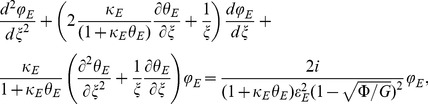
(21)


The corresponding dimensionless boundary conditions are: 
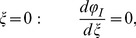
(22)

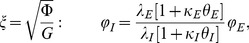
(23)

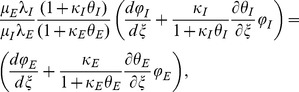
(24)

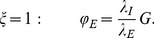
(25)


### Dimensionless thermal model

In the same manner, we can use the dimensionless variables and parameters in order to obtain the following dimensionless thermal model:

Therefore, the internal phase equation can be written as: 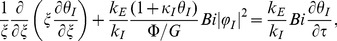
(26)and external phase as: 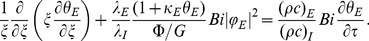
(27)


The associated boundary and initial conditions are given by: 
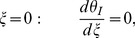
(28)

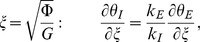
(29)

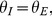
(30)

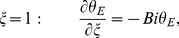
(31)

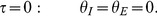
(32)


The system of equations to be solved is formed by Eqs. (20) and (21) for the current density distribution coupled through the boundary conditions (23) and (24); and Eqs. (26) and (27) for the thermal behavior also coupled through their boundary conditions (29) and (30). It should be noticed that there is a coupling between the electromagnetic and thermal model due to the dependence of resistivity with temperature which is expressed by 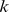 parameter, henceforth the coupling parameter.

## Solution Methodology

The above dimensionless electromagnetic and heat conduction equations, together with their boundary and initial conditions, represented here by the system of Eqs. (20)–(32) was solved by using a conventional iterative finite-differences method [19].

The electromagnetic equations are given by the system of Eqs. (20)–(25). These equations are complex because the right-hand sides of Eqs. (20) and (21) include, as a factor, the imaginary number 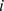. Therefore, we separate for each region the electrical current density 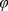, in a real part, 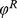, and an imaginary part 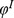, through the relationship 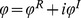. The resulting equations are discretized together with the boundary conditions (22)–(25) considering central differences. In this form, we can construct a matrix system which can be solved with a simple Gauss elimination method.

The corresponding equations for the thermal model given by the set of Eqs. (26) and (27) together with the boundary and initial conditions (28)–(32), require a different treatment. In this case, the above equations represent a non-stationary problem. Therefore, the numerical procedure is based on the well-known Crank-Nicholson finite difference scheme. In this manner, we obtain a tridiagonal matrix which is solved by the tridiagonal matrix algorithm (TDMA), also known as the Thomas algorithm.

Finally, we introduce the following iterative scheme: firstly, a uniform profile for the temperature is considered, then we solve for the electrical current density. In this manner, we can obtain the modulus or absolute value of this function. Introducing the above result into Eqs. (26) and (27), we obtain the first nonuniform temperature profile. Again, we can obtain a new current density and the foregoing procedure is repeated until a convergence criterion is fulfilled. This criterion is based on the comparison of the temperature and current density profiles.

## Validation

It is important to note that if the coupling parameter is equal to zero (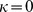), the equations associated with the electromagnetic behavior, Eqs. (20) and (21), are no longer affected by the temperature; thus, the system can be solved analytically. The solutions for the current density distributions, 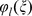 and 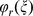 (liquid and rock phases, respectively) are: 
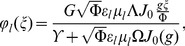
(33)and 
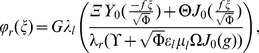
(34)where 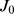, 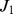, 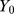, 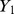 denote the Bessel functions of first and second kind and of zeroth and first order, respectively. The variables 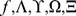 and 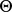 are defined as: 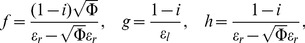
(35)

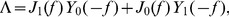
(36)

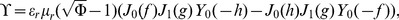
(37)

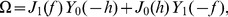
(38)

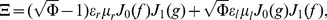
(39)

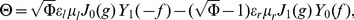
(40)


**Figure 2:**
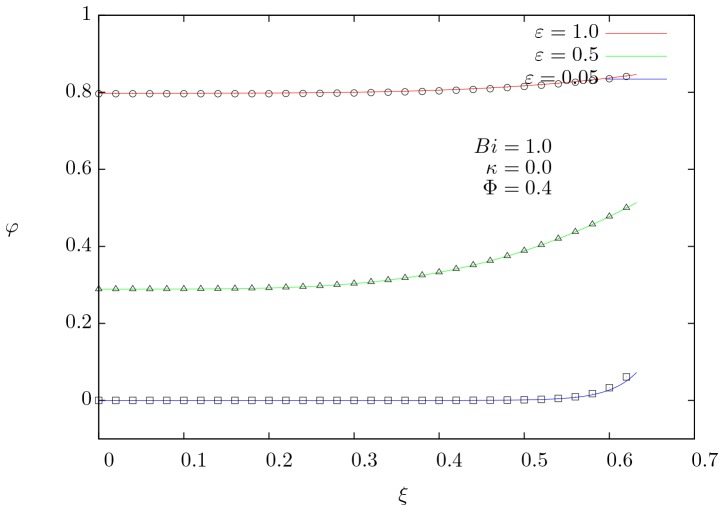
Dimensionless current density distribution 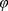 as a function of the radial coordinate 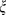 for both conducting phases and three different values of the skin parameter 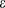. Solid lines are computed from the analytical solution, the open symbols represent numerical simulation results.

This analytical solution has great relevance since it serves as a validation test for our numerical results. As shown in Fig. 2, the numerical simulations (open symbols) agree very well with the analytical solutions (solid lines).

## Results and Discussion

In the system of equations (26)–(32) we have two coupling parameters 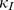 and 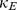. They, however, depend on each other because they are affected by the same 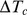, thus 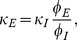
(41)therefore it is necessary to know only one parameter. We choose the level of coupling between thermal and electrical model as 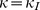.

In the same manner the skin parameter for the internal phase, 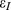, is related to that of the external one, because both phases transmit a wave at the same frequency. Thus we have: 
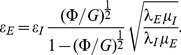
(42)


From now on, 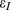 will be simply called 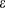, the depth of penetration of the electrical signal. Therefore, the main parameters in this problem are: 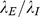, 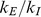, 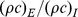, 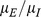, 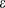, 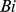, 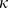 and 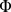.

To study the transmission of the electrical signal in a composite medium, we perform a parametric analysis based on the following parameters: 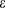, which is a function of the frequency, the ratio of resistivities, 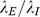, and the ratio of thermal conductivities, 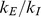. For simplicity, all these calculations assuming 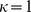, 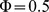, 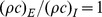, 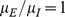 and 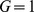; also, we consider 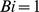, which assures that the heat is efficiently transferred by convection from the external phase to the environment. To assure steady state solutions we performed all calculations using 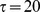.

### Effect of 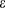


**Figure 3:**
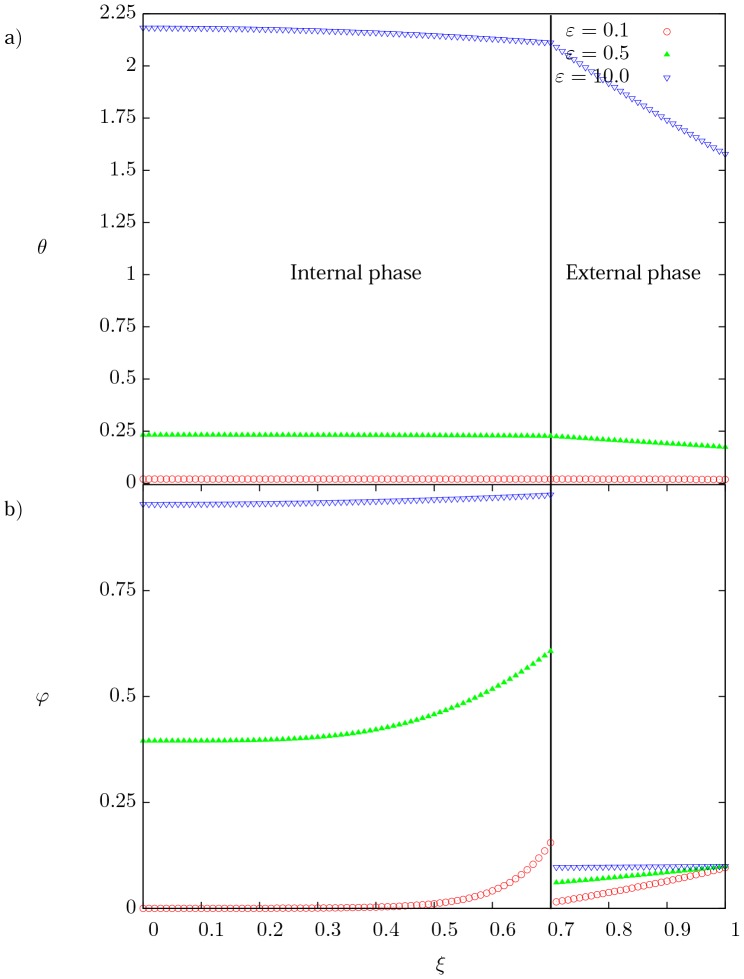
Spatial profiles of temperature and current density. (a) Dimensionless temperature distribution, and (b) dimensionless current density distribution as a function of the dimensionless radial coordinate for three different values of the skin parameter 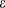. For 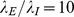 and 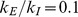.

To analyze the effect of the skin parameter on the current density and temperature, numerical results were obtained from three different values of 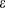 (0.1, 0.5 and 10) and are presented in Fig. 3. The series of three temperature profiles shown in Fig. 3a represents the steady-state solution for each corresponding value of 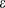. For lower values of the skin parameter (which correspond to electric signals with high frequency), the temperature profiles become more uniform in both phases; on the contrary, when grows a slightly parabolic temperature profile is exhibited in the internal phase and even a steeper parabolic profile is observed for the external one. This parabolic behavior comes from the Joule effect (source therms in equations (26–27)). Figure 3b shows the current density distribution as a function of the dimensionless radial coordinate for the same conditions shown in Figure 3a. Clearly, for small values of 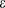, the skin effect becomes noticeable in both phases. In particular the current density in the internal phase is higher in regions close to the interface (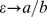). On the opposite side, high values of 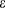 show nearly constant current distribution in both phases, hence the electric power tends to be transmitted as direct current.

### Effect of 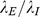


**Figure 4:**
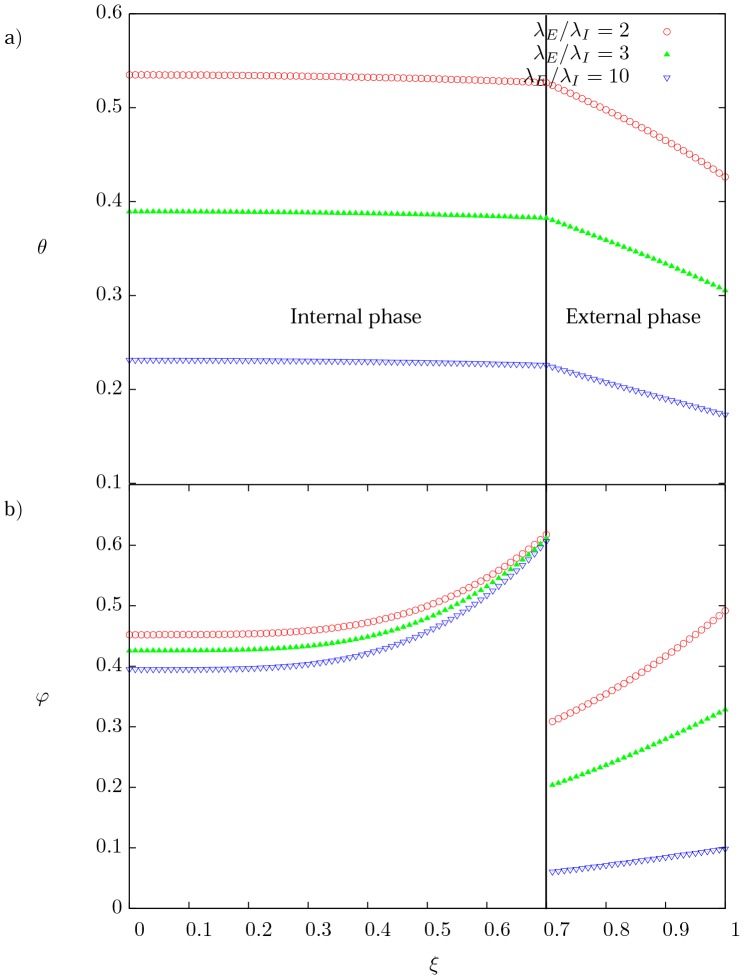
Spatial profiles of temperature and current density. (a) Dimensionless temperature distribution, and (b) dimensionless current density distribution as a function of the dimensionless radial coordinate for three different values of the ratio of resistivities 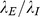. For 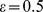 and 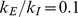.

Figure 4a shows the dimensionless temperature and Figure 4b current density distributions for three different values of the ratio of resistivities 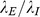 (2, 3 and 10). For smaller values of the 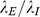 ratio, it is expected that most of the current will flow through the internal phase which also increases the temperature due to the heat generated by Joule effect. In constrast, when the ratio 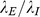 tends to increase, the current density profiles in the external phase are redistributed at the outer surface showing a more pronounced skin effect and smaller current densities gradients. The shape of the temperature profiles does not appear to change substantially. The temperature profile in the external phase becomes slightly parabolic in shape and its negative slope is less pronounced indicating smaller temperature gradients.

### Effect of 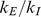


**Figure 5:**
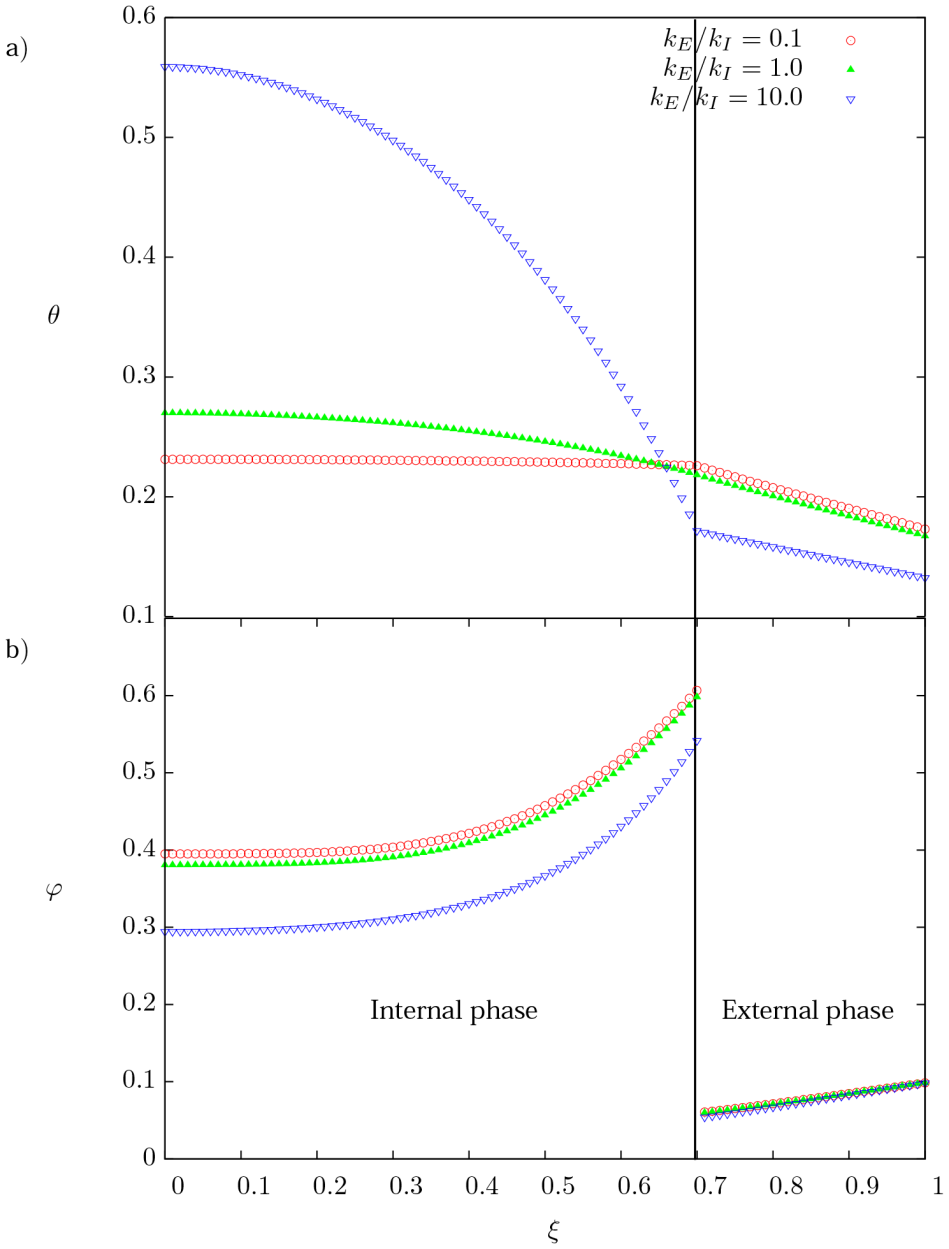
Spatial profiles of temperature and current density. (a) Dimensionless temperature distribution, and (b) dimensionless current density distribution as a function of the dimensionless radial coordinate for three different values of the ratio of thermal conductivity 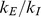. For 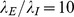 and 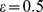.

Figure 5a shows the dimensionless temperature distribution for three different values of the ratio of thermal conductivities 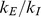 (0.1, 1.0 and 10). High 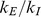 ratios imply an excellent heat transfer by diffusion in the external phase compared with the internal one, consequently the heat generated at the internal phase faces more resistance to be transferred to the external one, which leads to a higher temperature gradient, while the external phase transfers the energy to the environment optimally. For the case when 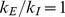 both media behave thermally as a single phase. On the other hand, when the ratio 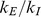 is small the internal phase transfer the heat generated in a better way and as a result we obtain a flatter profile than in the external phase. Figure 5b shows the current density distribution under the influence of thermal conductivities (above mentioned). The electromagnetic equations (20) and (21) are affected by temperature gradients, as a result, the higher temperature gradient the more affected the current density profile; for the external phase where the temperature gradient is basically the same, the current density is almost not affected.

## Experimental Validation

The measurement of the electrical conductivity of porous media has applications in many areas of science and technology such as geothermal reservoir engineering and soil science and is particularly important in the oil and gas industry to estimate the amount of petroleum in reservoirs. The main mechanism for electrical transport in porous rocks and soils is electrical conduction through the water filling the pore space. Furthermore, the pore-scale geometrical features of rocks and soils have a significant effect on their bulk electrical conductivity [20]. Conversely, the electrical conductivity can be used to infer the characteristics of pore space in rocks and soils [21].

The previous developed model can be used to study electromagnetic transmission through rocks; which are a type of natural composite medium with two interpenetrating, percolating phases: the pore and solid networks [2]. It is worthy of note that rocks are also clasified as porous media [22].

For the present application we suppose that the solid phase consists of a rock matrix forming a solid cylinder (external phase). The fluid phase, typically a brine solution, represents a static incompressible fluid filling a pore (internal phase) which is embedded into the solid phase. From now in advance, all the variables with subindex 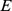 will be rename with the subindex 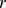 which stands for rock domain while for 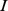 we will use 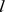, liquid domain.

The relationship that links the electrical resistivity of a rock to its porosity was empirically determined by Archie [23]. He proposed that the formation factor depends on porosity obeying an inverse power law: 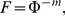
(43)where 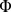 is the bulk porosity and 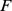 is the formation factor, defined as the resistivity of a porous medium completely saturated with a conductive liquid divided by the liquid resistivity. The parameter 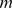 is estimated by a fitting regression analysis of experimental data and is often called the cementation exponent; it implicitly provides information about the pore structure.

Since its publication in 1942, Archie's law has been widely used in the petroleum industry to characterize reservoirs. This fact is easily explained by two aspects. First of all, the formula is simple and compact, which are undoubtedly attractive features. Secondly, this law describes to some extent the experimental trends of data from many different types of rock samples [24], [25]. Nevertheless, it should be noted that the relation between 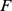 and 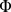 data acquired from reservoirs commonly show considerable scattering [24]–[26], which strongly suggests a non-trivial relationship between these variables. Therefore, Archie's law is simply a crude approximation.

In view of the previous definition for 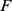 and from the set of Eqs. (20)–(25), the following expression is constructed: 
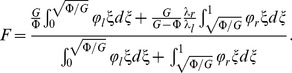
(44)


In the previous equation 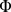 is known as porosity, which previously was defined as volumetric fraction and now the tortuosity, 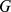, is calculated according to the expression reported by Maciej [27]

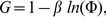
(45)where 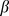 is a parameter which relates the porosity with tortuosity.

Typically in a porous medium applications 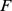 is shown as function of 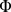 in log-log plots, showing a negative slope behavior (power-law dependence) [23]. It is worth pointing out that for the present model we fix the values for the thermal and electrical properties of rock and brine, and also an initial value for 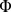 (which is increased at a constant 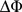); now we calculate 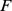 from equation (44) for each value of 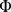.

**Figure 6:**
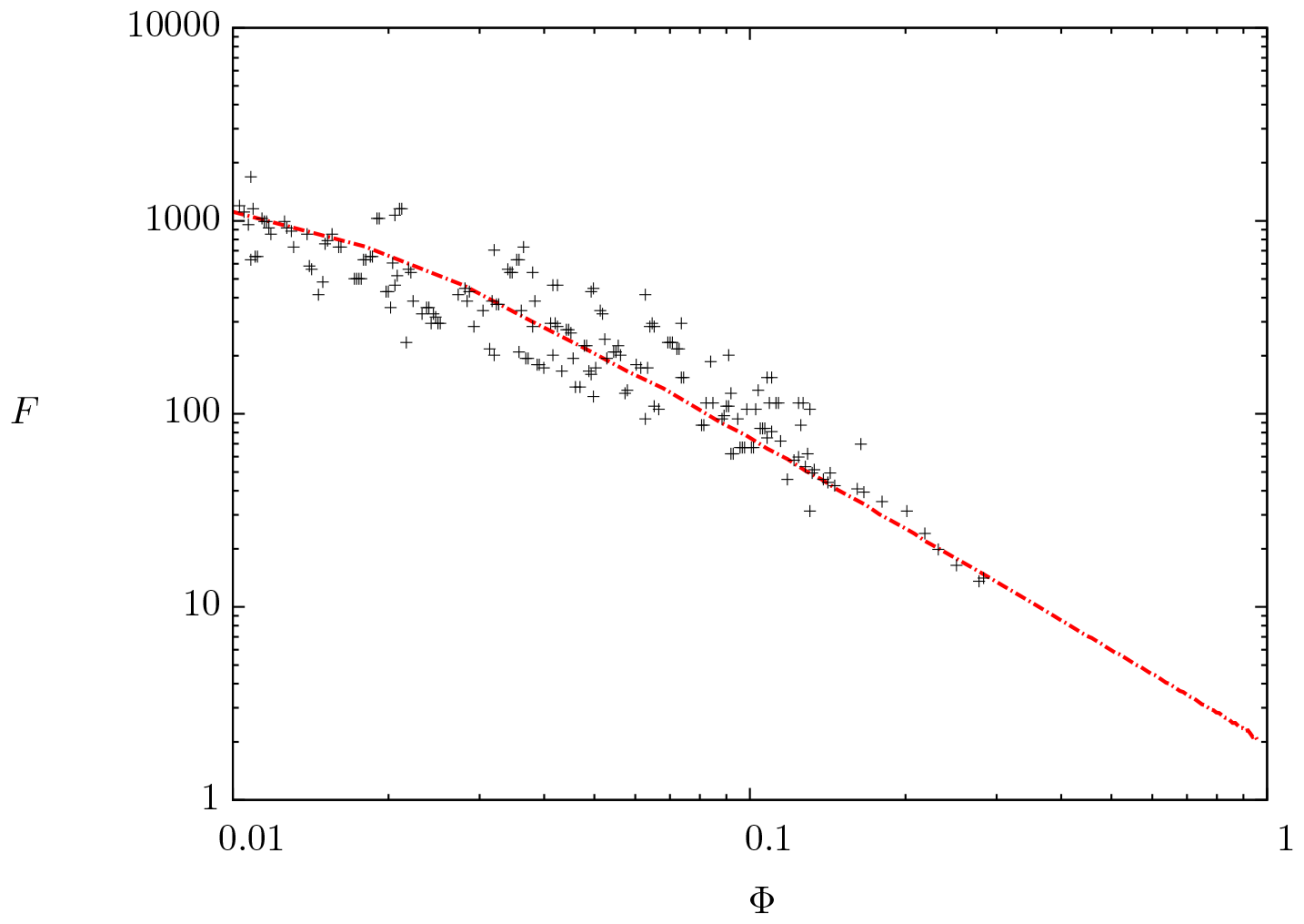
Comparison between the mathematical model (dashed dotted line) and experimental data (crosses symbols) taken from [28]. All numerical results were computed with: 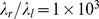, 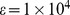 and 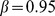.

**Figure 7:**
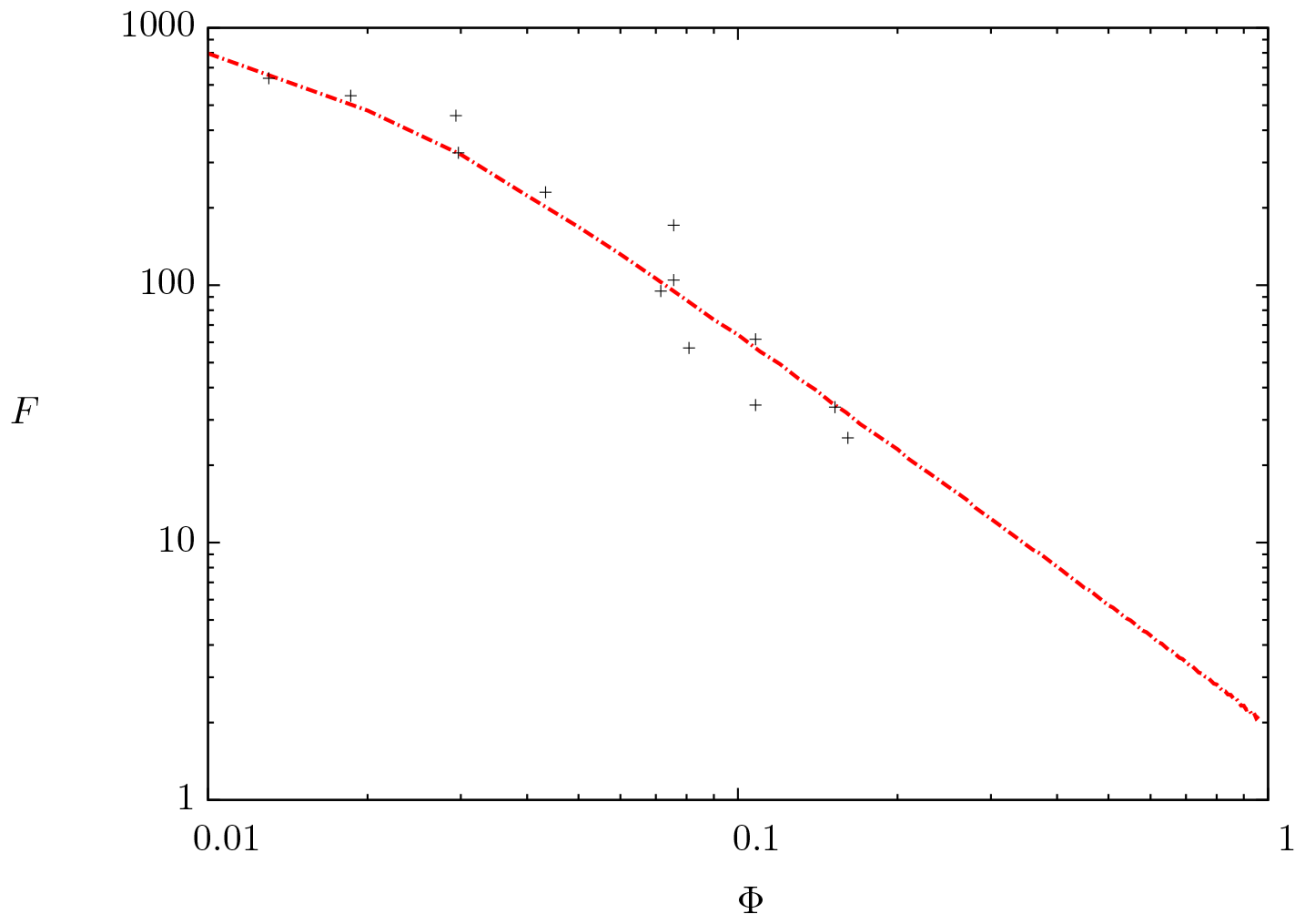
Comparison between the mathematical model (dashed dotted line) and experimental data (crosses symbols) taken from [29]. All numerical results were computed with: 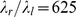, 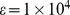 and 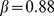.

Figures 6 and 7 show the formation factor as a function of porosity. The prediction of our model is compared directly with experimental data [28] and [29]. The numerical model represented by the dashed dotted line is solved with typical values of electrical resistivity for both phases [30]. The bulk magnetic permeability of the rock phase is of the same order than that of the liquid [31]. Moreover, the skin parameter was taken 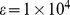, which involves frequencies in the range from 1 to 1000 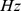 and the penetration of the electromagnetic wave in the whole domain for which experiments are usually conducted [32]. A remarkable agreement is observed between the numerical results and the experimental data. The model accurately reproduces the typical decreasing dependence of 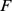 with 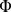 for the experimental data; clearly, both the experimental data and our model for the formation factor do not follow a power-law dependence on porosity as proposed by Archie [23].

## Conclusions

A nonlinear theoretical model that describes the combined effect of heat transfer and transmission of an electromagnetic wave through a composite medium was developed. The model reveals the influence of the properties of the composite medium and the electrical signal on the current density and temperature as a function of the radial coordinate. Basically three parameters were varied: the skin depth for which the well-known skin effect is clearly observed for electric signals of high frequency; the ratio of electrical resistivities, showing different current distribution and their effect on the temperature through Joule's effect and finally the ratio of thermal conductivities which shows that the current density is clearly affected by temperature gradients.

Additionally, our model allows the calculation of the formation factor without the use of empirical relations, such as Archie's law. Now, the formation factor can be calculated directly if the physical properties of the porous media and the electric signal are known. A curve of the formation factor as a function of the porosity shows a similar trend than that found in experimental data for a rock porous medium, clearly the behavior is non-linear and is well predicted by our model in strong contrast with the linear log-log Archie's law. To our knowledge, such a theoretical model does not exist in the specialized literature.
